# Editorial: Pathophysiology of Chronic Kidney Disease and Its Complications, Second Edition

**DOI:** 10.3390/biomedicines14020432

**Published:** 2026-02-14

**Authors:** Yuji Oe

**Affiliations:** Department of Nephrology, Graduate School of Medicine, Tohoku University, Sendai 980-8574, Japan; yuji.oe.b3@tohoku.ac.jp; Tel: +81-22-717-7163

The number of patients with chronic kidney disease (CKD) is increasing, and CKD is a major risk factor for progression to end-stage kidney disease (ESKD) requiring renal replacement therapy. Moreover, it affects multiple organ systems and causes cardiovascular complications, thereby worsening prognosis and quality of life [1,2]. This Special Issue aims to shed light on the pathogenesis and inter-organ crosstalk involved in CKD and explore new biomarkers and therapeutic strategies that may benefit patients with the disease. It comprises eight manuscripts—four original research articles and four review articles. The following is a brief summary of the main findings presented in each contribution.

Although serum creatinine, which reflects glomerular filtration, is considered the gold standard among renal function markers, it has limitations, particularly its inability to detect early kidney injury. Moreover, serum creatinine levels can be influenced by factors such as muscle mass and dietary protein intake [3]. Therefore, there is growing interest in exploring alternative or complementary biomarkers for CKD. In this context, urinary kidney injury molecule-1, which reflects tubular damage, and soluble tumor necrosis factor receptors, which serve as inflammatory markers, have been proposed as potential biomarkers of CKD [4]. To shed light on this, Gervasini et al. demonstrated the prognostic value of amino acid profiling in patients with CKD [5]. They reported that, among the 28 amino acids examined, citrulline and the tryptophan metabolic route to kynurenine are promising biomarkers and that factoring these into CKD risk assessment can improve the accuracy of existing prognostic prediction methods, such as serum creatinine [5].

It is well known that vitamin D deficiency and insufficiency are common among patients with CKD, particularly those undergoing hemodialysis (HD), and that these conditions are often treated with vitamin D supplementation [6]. Active vitamin D analogs are recommended for managing CKD-related mineral and bone disorders in patients with advanced CKD or those undergoing dialysis. However, the efficacy of native vitamin D supplementation based on serum 25-hydroxyvitamin D (25(OH)D, calcidiol) levels remains controversial [7,8]. Tarasewicz et al. demonstrated that high-dose cholecalciferol administered during dialysis for 9 weeks safely and effectively increased 25(OH)D levels in patients on HD [9]. It also increased 1,25(OH)_2_D levels without significantly affecting parathyroid hormone levels. Further large-scale, long-term studies evaluating the effects of this drug on vitamin D, calcium, and phosphorus metabolism could provide useful insights for improving the quality of life of patients on HD [9].

Omega-3 fatty acids have been shown to lower triglyceride levels and reduce cardiovascular risk, and evidence suggests that they may help prevent the onset of CKD. Their multifaceted mechanisms of action have attracted considerable attention [10,11]. Choi et al. investigated the organ-protective effects of omega-3 fatty acids from the perspective of mitochondrial health [12]. Using an adenine-induced CKD model, they demonstrated that these fatty acids upregulated PGC1α and PINK1 expression in the heart and kidneys, and normalized DRP1 expression and mitochondrial DNA content in the heart. Thus, omega-3 fatty acid treatment may help maintain mitochondrial homeostasis by promoting mitochondrial biogenesis and PINK1-dependent mitophagy in CKD [12].

Hyperbaric oxygen therapy (HBOT), the administration of 100% oxygen at an elevated atmospheric pressure to enhance oxygen delivery to tissues, is widely used to treat various clinical conditions, ranging from acute diseases such as decompression illness, gas gangrene, necrotizing fasciitis, burn injury, and carbon monoxide or cyanide poisoning, to chronic disorders where it promotes tissue regeneration, including problem wounds [13]. Beyond these conditions, recent studies have demonstrated the beneficial effects of HBOT in CKD, such as diabetic nephropathy [14]. Vukovic et al. used a 5/6 nephrectomy rat model of CKD to evaluate the effects of HBOT [15]. They also examined the combined effects of apocynin, an NADPH oxidase inhibitor that exerts renoprotective effects in CKD [16]. Both apocynin and HBOT improved hypertension and histological damage in rats with CKD, and the combination therapy ameliorated renal impairment to a similar extent. Further studies are needed to clarify the underlying mechanisms, particularly the effects of these treatments on fibrotic responses and hypoxic conditions [15].

Physical inactivity worsens outcomes in patients undergoing HD, whereas exercise offers benefits through mechanisms that remain unclear [17]. There is growing interest in the role of microRNAs (miRNAs) in regulating gene expression under various disease conditions, and they may mediate the effects of physical activity [18]. Elia et al. designed a 3-month case-control study to assess exercise-induced changes in miR-9 and miR-30b, which are associated with vascular calcification, in patients on HD [19]. They presented the study protocol along with a useful brief review of miRNAs and physical activity in this population [19].

Protease-activated receptors (PARs) are a family of seven-transmembrane G protein-coupled receptors that become activated by coagulation proteases and are involved in the pathogenesis of various kidney diseases [20,21,22]. We reviewed the role of PAR2, one of the four PAR family members [23]. PAR2 is abundantly expressed in the kidney, and its expression increases under disease conditions. Although both harmful and protective roles of PAR2 signaling in kidney injury have been reported, the signaling generally promotes proinflammatory and profibrotic responses, suggesting that PAR2 could be a novel therapeutic target for CKD [23].

In addition, two comprehensive review articles have been published. Heart failure with preserved ejection fraction (HFpEF) is a heart failure syndrome in which the left ventricular ejection fraction is preserved, but impaired myocardial relaxation leads to elevated filling pressures and typical heart failure symptoms, and it frequently coexists with chronic kidney disease [24]. Bonacchi et al. provided deeper insights into the complex pathophysiological relationship between HFpEF and CKD, reviewed the renal effects of heart failure therapies, and proposed a practical step-by-step algorithm to guide the optimal use of these treatments in clinical practice [25]. Diabetic kidney disease (DKD) is a leading cause of CKD and a major risk factor for progression to ESKD. From 2000 to 2015, the global annual incidence of ESKD among patients with diabetes increased, with the prevalence rising from 19.0% to 29.7%, highlighting DKD as an increasingly critical health challenge worldwide [26]. Jha et al. provided an updated overview of DKD pathophysiology, renal injury biomarkers, and emerging treatment options—including SGLT2 inhibitors, GLP-1 receptor agonists, and novel drug candidates under development—offering readers a clearer understanding of recent advances in the field [27].

In conclusion, the collected papers cover a wide range of topics, from pathophysiology to biomarkers and the development of new treatments, which will help advance our understanding and management of CKD. Finally, as the editor of this Special Issue, I would like to express my sincere gratitude to all contributors for their valuable work and dedication.
